# Head and Neck Porocarcinoma: SEER Analysis of Epidemiology and Survival

**DOI:** 10.3390/jcm11082185

**Published:** 2022-04-14

**Authors:** Matteo Scampa, Rastine Merat, Daniel F. Kalbermatten, Carlo M. Oranges

**Affiliations:** 1 Department of Plastic, Reconstructive and Aesthetic Surgery, Geneva University Hospitals, University of Geneva, 1205 Geneva, Switzerland; matteo.scampa@hcuge.ch (M.S.); daniel.kalbermatten@hcuge.ch (D.F.K.); 2 Dermato-Oncology Unit, Division of Dermatology, Geneva University Hospitals, University of Geneva, 1205 Geneva, Switzerland; rastine.merat@hcuge.ch

**Keywords:** SEER, survival, porocarcinoma, malignant, poroma, epidemiology, treatment

## Abstract

Porocarcinoma is a rare malignant adnexal tumor. Little is known about the location of the disease in the head and neck. Our aim is to offer the largest analysis of demographic, pathological, and treatment patterns of head and neck porocarcinoma in comparison with other locations of the neoplasm from an epidemiologically representative cohort. Method: The Surveillance, Epidemiology, and End Results program of the National Cancer Institute was searched for all cases of porocarcinomas diagnosed between 2000 and 2018. This database is considered representative of the US population. Demographic, pathological, and treatment variables were compared between the head and neck and other regions. Overall and disease-specific survival was calculated and compared between groups. Results: 563 porocarcinomas were identified, with 172 in the head and neck. The mean age was 66.4 years. Males were more affected in the head and neck. Regional and distant invasion rates were low (2.9 and 2.3%, respectively). Local excision and Mohs surgery were the most frequent therapies. Five-year overall survival was 74.8%. Five-year disease-specific survival was 97%. Conclusions: Head and neck porocarcinoma affects more males than females. Regional or distant metastatic rates are low and overestimated in previous literature. Disease-specific mortality is low. Surgery remains the mainstay of treatment.

## 1. Introduction

Adnexal tumors are rare neoplasms developing from sweat glands, hair follicles, or sebaceous glands. They include benign and malignant entities. Porocarcinoma is a malignant tumor arising from eccrine sweat glands, first described in 1963 by Pinkus et al., with an incidence as low as 0.05 to 1.8 per 100,000 person years [1,2,3,4,5]. It affects mainly the elderly population [6]. Lower limb porocarcinoma typically presents as a plate arising from a previously known eccrine poroma. However, nodular or verrucous aspects associated with possible ulcerations have also been described [7,8]. Atypical forms can exist, for which the presumptive diagnosis is often related to more common skin malignancies such as squamous cell carcinoma or extra-mammary Paget disease [7,9]. Although dermoscopy may help orientate diagnosis, biopsy with histopathological analysis is necessary to affirm it [6]. Porocarcinoma presents diverse histological patterns necessitating immunohistochemical staining such as CEA or EMA to distinguish it from other neoplasms [6]. Due to the high local recurrence and potential to metastasis described in the literature, porocarcinoma has been presented as an aggressive neoplasm [10]. However, recent studies suggest that cutaneous adnexal tumors have an elevated age-adjusted relative survival and disease-specific survival [2,3,4,11]. Controversies regarding the duration of the development process exist, with porocarcinoma pathogenesis not being fully understood. Studies suggest it could arise de novo or degenerate from a benign poroma in a process of several years [12]. No global consensus on the treatment of porocarcinoma exists to our knowledge due to scarcity of evidence.

The head and neck and lower extremities are the regions most affected by porocarcinoma [4,13]. Due to aesthetic concerns and proximity with functional organs such as the eyes or mouth, the head and neck is a region where surgical treatment is associated with technical challenges requiring close collaboration between dermatologists and plastic surgeons to offer the best excision and reconstruction strategy. In order to do so, good knowledge of tumor behavior and epidemiology is essential. Most of the data concerning head and neck porocarcinoma come from meta-analysis, including small case series and case reports [9]. The aim of this study is to offer recent and reliable data on epidemiologic characteristics of head and neck porocarcinoma and analyze the different treatment patterns by including cases from a single database. The Surveillance, Epidemiology, and End Results (SEER) program of the National Cancer Institute is chosen because it reports data in a comprehensive way and is considered representative of the U.S. population. It offers the benefit that all patients are classified and characterized under the same rules inside the cohort. We aim to offer data that will help guide therapeutic decisions and future research on this rare neoplasm.

Studies on porocarcinoma based on SEER data have been conducted, but none of them focused specifically on the head and neck and none highlighted possible differences in tumor characteristics between this region and other localizations [2,11,14].

## 2. Materials and Methods

Patient selection: The SEER program was searched across 18 different registries for all porocarcinoma cases (8409/3 eccrine poroma, malignant) between 2000 and 2018. Data were extracted from the survival section of the National Cancer Institute SEER*stat software (seer.cancer.gov/seerstat) version 8.3.9 and multiple variables were analyzed using IBM SPSS version 27 (IBM, Armonk, NY, USA). No ethical board approval was necessary because the SEER program provides open-access de-identified data.

Statistical analysis: Variables were compared between the head and neck and other localizations using the T-test or Chi-square test where relevant. Survival analysis was conducted for the head and neck localization. We calculated overall survival (OS) using the Kaplan–Meier method, and then disease-specific survival (DSS). The use of DSS was justified by evidence suggesting that porocarcinoma might be a slow-evolving tumor of the elderly, where death from other causes might be frequent. The log-rank test was used to compare survival between different variable values. A *p*-value inferior or equal to 0.05 was considered statistically significant.

Variable selection: Only SEER-defined variables were used in this study. For some variables that contain multiple values such as “surgical therapy”, similar therapies were merged in subgroups to ease the interpretation and avoid single patient values. In the stage variable, localized disease was defined as a lesion confined to the dermis and subcutaneous tissue. Regional disease was defined by extension to local structures (bone, cartilage, muscles) and/or regional lymph nodes. For the head and neck, perineural or skull foramen invasion was also defined as regional disease. Distant disease was defined as metastasis, distant lymph node invasion, or distant site without contiguous extension. For the eyelid, more specific definitions were given: Localized disease must be limited to the eyelid but can affect the tarsal plate and underlying orbicularis muscle. Regional was also defined as an extension outside the eyelid with or without ocular structures affected. Distant disease had a stricter definition, with the nasal cavity, naso-lacrimal duct, and sinus considered metastasis, even with contiguous extension [15]. Tumor size was extracted from two SEER variables covering different time periods. Size was reported in mm from 1 to 200 mm, but some cases lacked precision and were reported as ranges or as “bigger than”. Those cases were censored (six cases in total).

## 3. Results

A total of 563 cases of porocarcinoma were identified, of which 172 (30.6%) affected the head and neck region (Table 1). The skin of the lower limb and hip was the most affected region in this population, with 190 cases (33.7%), followed by the skin of the head and neck (30.6%) and then the skin of the trunk (19.5%).

**Figure 1:**
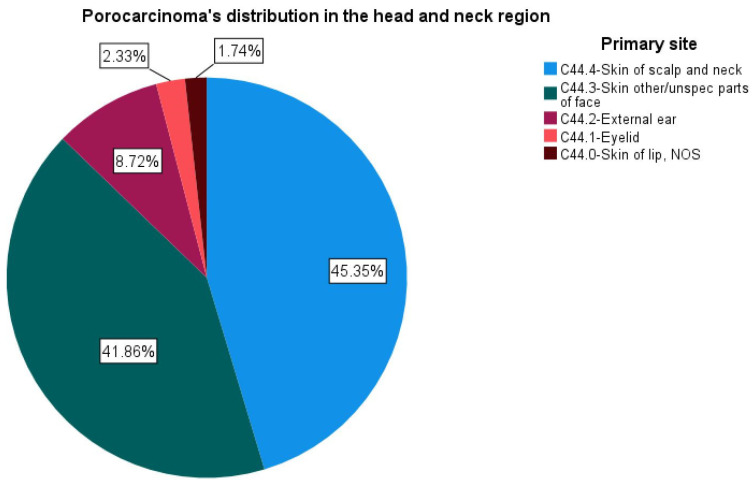
Porocarcinoma distribution in the head and neck region.

In the head and neck region, C44.4—Skin of scalp and neck was the most reported location, with 78 cases, followed by C44.3—Skin other/unspec parts of the face (72 cases). (Figure 1).

In the head and neck region, mean age at diagnosis was 66.4 years old (σ = 17.6), with a range from 10 to 98 years. The median age was 69 years and was similar for the overall population, the head and neck, and other locations. White race was predominant (86%). Males were more affected in the head and neck (64%) compared to other locations (51.2%) (*p* < 0.05).

Pathological grade was reported sporadically and could not be interpreted. Disease was diagnosed mostly at a localized stage in the head and neck region (69.8%), and distant disease remained rare (2.3%). For 25% of the tumors the initial stage was unknown. Tumor size in this region was reported for 72 cases, ranging from 2 to 85 mm, with a mean value of 18.8 mm (σ = 18.3) and a median value of 13 mm.

Excision or destruction of the lesion was the most frequent surgical therapy in the head and neck region (30.8%) and in other localizations (31.5%). However, biopsy followed by gross excision (26.3%) and wide excision with margins over 1 cm (23.3%) was significantly more frequent in other localizations than in the head and neck. Mohs surgery (19.8%) was more represented in the head and neck region. Radiotherapy and chemotherapy were used rarely in all locations.

**Figure 2:**
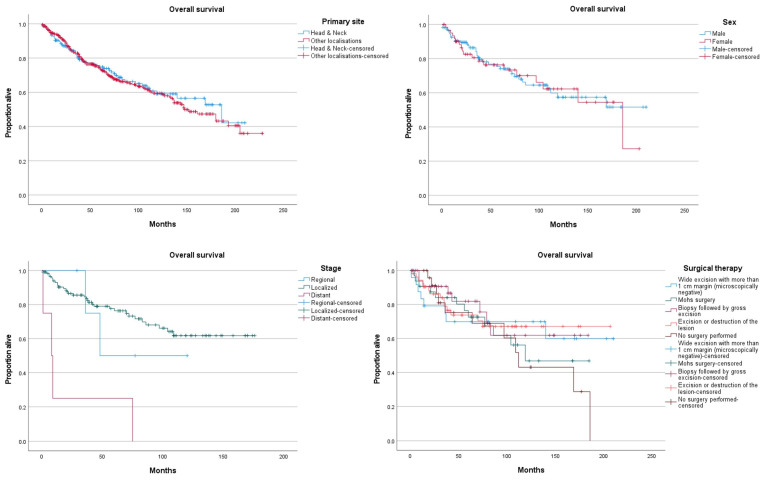
Overall survival curves of head and neck porocarcinoma according to sex, stage, and surgical treatment.

Mean overall survival in the head and neck region was 138.9 months (95%CI {124–153.8}), with 74.8% alive at 5 years. It did not differ significantly from other locations (Figure 2).

Five-year DSS for the head and neck was 97%, with only four patients out of 172 declared dead from porocarcinoma. A DSS comparison between variables was not realized.

In the head and neck region, overall survival did not differ significantly between male and females. OS did not differ significantly between races. Distant disease was associated with lower OS than localized disease (*p* < 0.05), but differences in survival between localized and regional disease did not differ significantly. Surgical therapies did not differ significantly in terms of OS. Radiation therapy use was associated with lower OS (*p* < 0.05). The use of chemotherapy did not significantly impact OS.

## 4. Discussion

Porocarcinoma is an extremely rare disease for which retrospective and prospective studies are not easily realizable due to the scarcity of cases. Most studies consist of small case series or case reports. An alternative for analysis of rare diseases is meta-analysis or registry-based studies such as ours. Despite limitations due to bias commonly encountered in registry-based studies, our work highlights the differences in tumor demographics and treatment patterns between the head and neck region and other localizations. This study is, to our knowledge, the biggest cohort focusing on head and neck porocarcinoma. Despite an important proportion of unknown data in certain variables such as pathological grade, chemotherapy, and radiotherapy that can jeopardize their interpretation, this study highlights some trends such as a low use of chemotherapy and radiotherapy in favor of surgery, a high rate of localized disease, and low mortality. It offers data on which further studies can rely to design more complete research protocols.

Compared to a finish registry analysis, our U.S. population was younger [4]. They reported the head and neck as the most affected region and found relative survival close to the general population [4].

Although porocarcinoma was found to be distributed equally between males and females in one of the biggest meta-analyses available [13], some studies described a female predominance [7,12]. On the contrary, we found a male predominance in the head and neck region that confirms Le et al.’s observation [9]. When assessing our full cohort with all locations, our male/female ratio was also close to 1. This could highlight a different pathogenesis between the head and neck region and other localizations. Exposure to chronic radiation and sunlight and association with other dermatologic diseases or immunosuppression are suspected to be risk factors for poroma and porocarcinoma development [13,16,17]. The white race seems more affected, which could be explained by an increased mutational charge in light skin phototypes due to UV radiation. This phenomenon might also explain the higher distribution of cases in the elderly population due to cumulative exposure. Patients under an immunosuppressive regimen for transplantation or immunodeficiency (HIV) are often affected by porocarcinoma [13,18]. If porocarcinoma arises from a poroma over several years, aesthetic concerns could lead to a lesion excision before malignant transformation, possibly explaining the lower incidence in females.

Interestingly, metastatic disease remained rare in our cohort compared to Salih et al.’s study, where rates of metastasis at diagnosis up to 30% were described [13]. This could be explained by a selection bias: Most of the meta-analysis data came from case reports and case series where advanced diseases are most likely reported compared to a localized successfully treated porocarcinoma. The proportion of localized diseases might have been higher, because for one fourth of the patients in our cohort the stage was unknown.

This study highlights a trend toward minimal invasive surgery such as Mohs surgery in the head and neck region compared to other regions. This result can be explained by aesthetic concerns and by the proximity of the organs in the face [19]. The scalp might be less subject to those concerns, but less invasive surgeries would still be preferred due to the difficulties associated with loss of soft tissues and the necessity of carrying out flap coverage in the elderly population. Because disease-specific mortality is low, concerns about recurrence in the elderly population, which is at higher risk from dying from other diseases, might favor a minimal excision of the lesion, explaining why excision or destruction of the lesion is the most frequent therapy.

No consensus on the best treatment for head and neck porocarcinoma exists to our knowledge due to scarcity of evidence. Mohs surgery seems to be a valid surgical strategy with promising results [20,21,22]. The role of regional lymph node biopsy is currently questionable. Storino et al. reported a low incidence of lymph node metastasis in patients without clinical adenopathy, whereas Tsunoda et al. reported higher rates of positive lymph nodes [23,24]. Poor evidence exists on the use of chemotherapy and radiotherapy, but radiotherapy could play a role in lymph node control [25,26].

Although wide resection, including sub-cutaneous tissues up to the fascial plane, and lymph node biopsy have been advocated by some authors [6,10,13], our results suggest that porocarcinoma might be less aggressive than initially suggested. De Giorgi made a similar observation in a smaller retrospective study in Italy, where mortality and tumor recurrence was low [27]. In this case, in selected patients, less invasive surgery could be favored to avoid morbidity linked to wide margins and lymph node procedures. Belin et al. suggested that histopathologic characteristics could help identify high-risk tumors where more invasive surgery would be needed [28]. The significant proportion of unknown data in this study and the high OS and DSS do not allow conclusions to be drawn on the superiority of one therapeutic option. Further studies are needed to assess the superiority of different therapeutical strategies.

Although metastatic disease remains low, it often requires systemic treatment. No recommendations on chemotherapeutic regimens exist, with various outcomes reported [29]. Little is known on the porocarcinoma mutational landscape, with HRAS and tumor suppressor gene mutations previously reported. Recently, studies identified fusions in the YAP-1 gene associated with poroma and porocarcinoma oncogenesis, which could be targeted by future immunotherapies [30]. Further research in this domain is required.

The high DSS highlights the fact that dying from porocarcinoma remains rare. It could be explained by slow growth in an overall elderly population that has higher chances of perishing from other causes. A limitation to this interpretation could be cases notified as dead from other causes, where that cause is an indirect consequence of the tumor or its treatments. However, OS remains high with 74.8% patients alive at 5 years, reducing the probability of this phenomenon.

Delays in diagnosis have been reported up to 5 years [13]. Because of indistinct clinical characteristics and potential slow growth, porocarcinoma incidence might be underestimated [7,27]. Interpretation of the effect of diverse variables such as staging and treatment is not feasible with DSS in this study because only a few patients died from porocarcinoma. Interpretation of differences in OS between variables should be approached cautiously because they are subject to multiple confounding factors in diseases with low mortality and lack data in certain variables. A future way to obtain valid data on the impact of those treatments would be a disease-free survival analysis that is not feasible in this cohort because relapse was not reported.

## 5. Conclusions

Head and neck porocarcinoma tend to affect more males than females compared to other areas. Our study suggests that regional or distant metastatic rates could be over-estimated in the current literature. Disease-specific mortality is low, and surgery remains the mainstay of treatment. Further studies assessing disease-free survival are required to determine superiority between different surgical therapies.

## Figures and Tables

**Table 1:** Demographic, pathological, and treatment characteristics of the study population affected by porocarcinoma.

	Head and Neck Skin N (%)	Other Sites N (%)	Statistical Difference *p*-Value	Overall N (%)
Age at diagnosis			*p* = 0.358	
Mean	66.4	67.9		67.4
Std deviation	(σ = 17.6)	(σ = 16.7)		(σ = 17)
Sex			*p* = 0.005	
Male	110 (64)	200 (51.2)		310 (55.1)
Female	62 (36)	191 (48.8)		253 (44.9)
Race			*p* = 0.092 *	
White	148 (86)	299 (76.5)		447 (79.4)
Black	8 (4.7)	34 (8.7)		42 (7.5)
Asian or Pacific Islander	7 (4.1)	29 (7.4)		36 (6.4)
American Indian/Alaska Native	0 (0)	1 (0.3)		1 (0.2)
Unknown ^ψ^	9 (5.2)	28 (7.2)		37 (6.6)
Grade			*p* = 0.612 *	
I: Well differentiated	3 (1.7)	10 (2.6)		13 (2.3)
II: Moderately differentiated	9 (5.2)	25 (6.4)		34 (6)
III: Poorly differentiated	13 (7.6)	20 (5.1)		33 (5.9)
IV: Undifferentiated; anaplastic	2 (1.2)	5 (1.3)		7 (1.2)
Unknown ^ψ^	145 (84.3)	331 (84.7)		476 (84.5)
Stage			*p* = 0.433	
Localized	120 (69.8)	265 (67.8)		385 (68.4)
Regional	5 (2.9)	15 (3.8)		20 (3.6)
Distant	4 (2.3)	4 (1)		8 (1.4)
Unknown ^ψ^	43 (25)	107 (27.4)		150 (26.6)
Tumor size			*p* = 0.081	
Mean	18.8 mm	24.4 mm		22.7 mm
Std Deviation	(σ = 18.3)	(σ = 24.7)		(σ = 23.1)
Surgery			*p* = 0.000	
No surgery performed	25 (14.5)	47 (12)		72 (12.8)
Excision or destruction of the lesion	53 (30.8)	123 (31.5)		176 (31.3)
Biopsy followed by gross excision	36 (20.9)	103 (26.3)		139 (24.7)
Mohs surgery	34 (19.8)	21 (5.4)		55 (9.8)
Wide excision with margins over 1 cm (microscopically negative)	24 (14)	91 (23.3)		115 (20.4)
Major amputation	0 (0)	4 (1)		4 (0.7)
Unknown ^ψ^	0 (0)	2 (0.5)		2 (0.4)
Radiotherapy			*p* = 0.841	
No/unknown	166 (96.5)	376 (96.2)		542 (96.3)
Beam radiation	6 (3.5)	15 (3.8)		21 (3.7)
Chemotherapy			*p* = 0.731 *	
No/unknown	170 (98.8)	385 (98.5)		555 (98.6)
Yes	2 (1.2)	6 (1.5)		8 (1.4)
Primary site				
C44.0—Skin of lip, NOS	3 (1.7)			3 (0.5)
C44.1—Eyelid	4 (2.3)			4 (0.7)
C44.2—External ear	15 (8.7)			15 (2.7)
C44.3—Skin other/unspec parts of face	72 (41.9)			72 (12.8)
C44.4—Skin of scalp and neck	78 (45.3)			78 (13.9)
C44.5—Skin of trunk		110 (28.1)		110 (19.5)
C44.6—Skin of upper limb and shoulder		79 (20.2)		79 (14)
C44.7—Skin of lower limb and hip		190 (48.6)		190 (33.7)
C44.9—Skin, NOS		8 (2)		8 (1.4)
C49.2—Connective, subcutaneous, other soft tissue: lower limb, hip		1 (0.3)		1 (0.2)
C51.9—Vulva, NOS		1 (0.3)		1 (0.2)
C60.9—Penis, NOS		1 (0.3)		1 (0.2)
C63.2—Scrotum, NOS		1 (0.3)		1 (0.2)

* Chi square model not valid (>20% cells with a count inferior to 5); ^ψ^ not included in chi square statistic. NOS: Not otherwise specified.

## Data Availability

Raw data available from the SEER program https://seer.cancer.gov/ (accessed on 7 January 2022).
